# Trust and distrust in interorganisational relations—Scale development

**DOI:** 10.1371/journal.pone.0279231

**Published:** 2022-12-16

**Authors:** Dagmara Lewicka, Agnieszka Freda Zakrzewska-Bielawska

**Affiliations:** 1 Department of Business Management, AGH University of Science and Technology, Krakow, Poland; 2 Department of Management, Lodz University of Technology, Lodz, Poland; Babes-Bolyai University, Cluj-Napoca, ROMANIA

## Abstract

Trust and distrust are considered as crucial elements in the management of hybrid interorganisational relationships with a view to helping to deal with their uncertainty and unpredictability. In this regard this paper seeks to conceptualize and clarify the interorganisational element of organizational trust and distrust and develop scales on which to measure it. The dimensions of the constructs have been tested on a sample of 400 respondents owners or top management on a representative sample by employment size. The research also attempts to identify the relationship between trust and distrust in inter-organisational relations. As a result of the research approach adopted, a one-dimensional scale for examining inter-organisational trust has been developed, as well as a two-dimensional scale for examining distrust in inter-organisational relations. The measurement scales developed and their validation conducted in this study represent a step forward towards the effective and reliable measurement of interorganisational trust and distrust. This is one of the few attempts at empirical verification of these constructs and the relationship between them, providing a comprehensive, operationally valid measure of interorganisational trust and distrust.

## 1. Introduction

Changes in business relations demonstrated in increasingly common relational contracts, outsourcing, strategic alliances, and networks have in recent years boosted interest in interorganisational trust [1–4]. On the other hand, many researchers have started calling for a more systematic approach to studying distrust in organisations [5, 6]. While being elements of social exchange, both trust and distrust seem to be key elements of both interpersonal and interorganisational relations [7]. They are simultaneously present in any type of relationship, which results from the fact that both categories are multi-dimensional and that many views about the same object may co-exist. As Cho [8] points out, both trust and distrust function in order to simplify the complexity of social phenomena. In Cho’s opinion, trust and distrust help decision-makers reduce uncertainty and vulnerability (i.e. risk) related to the consequences of decisions. Thus, both trust and distrust contribute to establishing and maintaining relations: trust through heightening the belief in positive intentions of the other party, and distrust through undertaking rational measures to secure transactions.

The scholars also point out that both trust and distrust have their dark and bright sides. At its essence, trust has a positive connotation and is regarded as a valuable relationship resource. However, doubts boil down to questions of whether there is an optimal level of trust and whether too high a level of trust is dangerous, causing a threat to the trust grantor and being an invitation to violation [9]. Excessive trust can be associated with a weakening of control and monitoring and, as a result, lead to opportunistic behaviour and impunity of the other party. The veil of close social relationships makes it easier to deceive the partner and postpone taking preventive or corrective action, resulting in greater losses [10]. Consequently, there is a notion of optimal trust, the level of which, conditioned by the situation, is difficult to define. However, according to Wicks [11], it should meet two generally defined criteria, i.e. to enable the achievement of objectives and to provide benefits such as cost savings and risk reduction.

In contrast, an analysis of research on distrust indicates that it has predominantly negative connotations. However, a number of authors emphasise positive or neutral consequences of distrust [6, pp. 9–13]. Among other things, they point to the possibility of making better decisions by enhancing the willingness to seek and consult information, increased sensitivity to cues indicating danger [6]. Another group of preventive activities related to distrust includes anticipatory actions to create organisational mechanisms and procedures to prevent violations [12]. In addition, the authors include among the bright sides of distrust an increase in the level of rationality of actions and effective self-regulation, hedging against the risk of opportunism, the limit of exploitation of exposed persons [6].

Certain tendencies can be noticed in the studies of trust and distrust. Firstly, the studies of trust are more popular than the studies of distrust; secondly, studies of both trust and distrust in the initial development stage pertained mainly to intra-organisational relations, and only as time passed, they focused more on interorganisational relations; and thirdly, irrespective of the development stage, the relationships between these two constructs were an important aspect of the studies. It is also worth drawing attention to the difficulties in studying trust and distrust and in synthesising the obtained results, due to the abundance of theories used to explain these phenomena [13]. This is corroborated by the analyses of several hundred papers on interorganisational trust, drawn up in such fields as economics, organisation and management theory, and sociology. In economics, trust is understood as being based on the calculation of perceived profits and losses. It is indicated that trust reduces or restricts ex ante and ex post opportunistic behaviours [14], thus contributing to considerable reduction of transaction costs and eliminating the need for constant judging of the other party’s credibility. On the other hand, distrust demonstrates the value of optimising transactions by means of reasonable and rational decisions. Similarly, in sociological theories, both trust and distrust are regarded as the following: relational, addressed to a specific recipient; and mutual, arising in the process of repeated transactions. Sztompka indicates that, “trust and distrust are specific bets regarding the future, uncertain actions of other people” [15, p. 310]. Despite the fact that this paper discusses interorganisational trust, it is trust and distrust that, in the opinions of sociologists, pertain mainly to the level of an individual, being based each time on human behaviour and decisions.

In organisation theories, trust is analysed as a strategic resource that allows an organisation to obtain a competitive advantage. Trust is treated as a rule, principle, or routine of an organisation indicating a constant decision-making pattern [16], which is activated also in the interaction with an external entity. Therefore, trust may be regarded as organisational practice [17]. As was pointed out, trust based on institutional foundations by means of credibility, efficiency, and binding rules complements relational trust in teams or networks [18].

Many authors indicate, however, that distrust is also useful in interorganisational relations, allowing the identification of possible threats and contributing to the risk mitigation. It is emphasised that the dynamics and ambivalence of the two constructs and their interrelationships are of great importance for exploring the nature of inter-organisational relationships, especially in the context of the management of cooperative relationships [6]. Summing up, it can be concluded that both trust and distrust play important roles in interorganisational relations, thus contributing to the balance and optimisation of decisions.

Quantitative research on trust has been conducted for at least seventy years, therefore there are many conceptualisations and operationalisations of this construct in the literature, which is due, among other things, to the diversity of research contexts adopted. This raises the question why is a new measurement scale for trust and distrust needed?

In response to this question it is worth pointing out that the intensive development of research in this area has resulted in a multitude of emerging scales characterised in effect by a lack of convergence and limited replicability [19]. However, what seems most significant, few of them dealt with inter-organisational trust. With regard to the scales examining trust in inter-organisational relationships, it should be noted that: (1) most of them were developed more than 20 years ago, (2) the vast majority of them examine trust as a unidimensional construct,(3) they are mainly concerned with the specific conditions of cooperation between organisations or in networks (4) they tested mainly the interpersonal components of trust.

Few of them were based on the assumption that inter-organisational trust should include both collective mental state and collective decision to rely on the trustor as the focal firm and the trustee as a partner organisation [20]. Since the publication of Seppanen and colleagues’ work critically reviewing quantitative research on trust [21], the inter-organisational relations research strand has developed considerably, e.g. in the late 1990s coopetition gained wide attention in the business community. This generated a need to revise approaches to trust and distrust in inter-organisational relationships.

In contrast, distrust in inter-organisational relationships, although it has recently received increasing interest from researchers, represents a very clear gap that needs to be filled both conceptually and in terms of measurement. Few scales for the study of distrust in inter-organisational relationships have been developed so far. Furthermore, there is little research on the interplay between trust and distrust in interorganisational relationships, which requires testing both trust and distrust. This is particularly relevant in the context of the development of research on different types of inter-organisational relationships, from which the study of trust and distrust should emerge as their relevant aspects [4].

What is missing, therefore, is a scale applicable to different types of inter-organisational relationships (dyad, triad, network) and between different entities, covering the components of inter-organisational trust identified in the literature and research at the collective organisational level.

Therefore, the aim of this paper is to develop scales to measure trust and distrust tested on the same research sample, as well as to identify the relationship between trust and distrust in interorganisational relationships. The measurement scale developed and validated in this study represent a step forward towards the effective and reliable measurement of interorganisational trust and distrust. To the best of the researchers’ knowledge, this is the first study to provide a comprehensive, psychometrically sound, operationally valid measure of these constructs and the relationships between them in relation to interorganisational relationships. The paper is structured as follows: after this introduction, the paper presents measurement scales used to study trust and distrust. To that end, the nature and importance of trust and distrust in interorganisational relations were presented. The main focus was put on the relationship between trust and distrust. Moreover, successive stages of construct development are presented, as well as the scale on which to measure it, and then a demonstration of its validity and reliability. In the final part of the paper, recommendations for the application of scales, limitation of scales, and directions for further research are discussed.

## 2. Interorganisational trust and its dimensions

Trust in interorganisational relations reduces the uncertainty and unpredictability that accompany mutual relations [22]. It allows organisations to face the complexity and changeability of economic conditions [23]. It is based on the gradable probability of behaviours and actions made by the other party (person or entity) in a specific situation. Therefore, the degree of trust is a decisive criterion in choosing between the multitude of alternatives in economic activity. Whenever action needs to be taken despite risk, trust becomes the basic strategy in coping with the impossibility to control the future. Moreover, it is indicated that trust is built in conditions of co-dependence and the expectation that the accepted vulnerability will not be expose [24]. In interorganisational relations, trust is regarded as an important element that allows not only a specific relation (or relations) to emerge between various entities, but also facilitates their development by reducing tensions and conflicts between partners as well as knowledge sharing, generating new knowledge and information disclosure. Trust has an influence on performance improvement and helps reduce opportunistic behaviours, at the same time contributing to the efficiency of collaboration between businesses [25]. It results from the fact that it is a tool for the self-control of partners and the coordination of joint actions [26]. Therefore, trust is seen as both an antecedent and an intangible resource stimulating the competitiveness of an enterprise [27].

Two contexts are indicated, wherein interorganisational trust is discussed: as a mental state and as a decision to trust, supported by specific behaviour. In the first approach, trust is regarded as an inclination or willingness to confide, wherein the aspect of voluntary dependence that is the foundation of trust is key [e.g. 28]. It is stressed in the literature that only individuals are able to place trust, but trust can be placed in both individuals as well as in teams and organisations. Despite the fact that an organisation cannot trust another organisation, their members may develop group orientation to trust a partner company based on interactions and institutionalisation. In this context trust concerns the expectancy aspect of an exchange partner’s behaviour and can be defined as a willingness to invest one’s resources in relations with another entity resulting from positive expectations that are a consequence of prior positive interactions [29]. Another group of definitions indicates decisions and related behaviours of the partner, as a result of which a business decides to rely on a different entity. It is assumed in such a situation that this decision and these actions are made collectively, which allows them to be treated as collective decisions made at the level of a specific organisational unit and resulting from the division of the orientation on trust among top management [30]. Therefore, the essence of interorganisational trust, as opposed to interpersonal trust, comprises both the collective mental state and collective decision to rely on the trustor as the focal firm and the trustee as a partner organisation [20]. In consequence, interorganisational trust is defined as the extent to which the members of a focal organisation trust the members of a partner organisation [31]. On the other hand, it should be stressed that trust originates with the trust of individual members of those organisations [32]. In this context, a definition may be quoted that covers both the aforementioned aspects by defining trust as expectation of a partner’s ability, goodwill, and self-relation in mutually beneficial activities that enable collaboration despite risk [33]. Most researchers treat interorganisational trust as a multi-dimensional construct, pointing to the existence of up to five dimensions such as credibility, benevolence, confidence, reliability, integrity, ability, dependability, responsibility, likeability, judgement, goodwill trust, contract trust, competence trust, fairness, reciprocity, predictability, and openness [21, p. 256]. In the above-mentioned review of scales for the study of interorganisational trust, researchers point out that there is no consensus as to the meaning of individual definitions; moreover, they are “blurred, overlapping, and even controversial” [34, p. 257].

The concepts indicating the multi-dimensionality of interorganisational trust are usually based on the model developed by Mayer et al. [28]. The model points to three fundamental components of trust: ability, referring to the partner’s skills, knowledge, and experience, i.e. their competence in cooperation; benevolence, which applies to caring for the common good and a positive atmosphere of cooperation; and integrity, or the level of respect for the mutually accepted principles for cooperation (the ABI model). The above conceptualisation is referred to, inter alia, by Gulati [35] and Zhong et al. [20], who regard trust in interorganisational relations as a two-element structure comprising goodwill and competence. Competence-based trust refers to the positive expectations of the partner’s ability to achieve goals. On the other hand, goodwill-based trust refers to the positive expectations of the partner’s goodwill, fairness, and mutual caring. As can be noticed, goodwill-based trust includes elements of integrity and benevolence, which in the opinion of the authors of the model, are difficult to distinguish by the respondents [20]. On the other hand, Blomquist [29], while conducting research on the asymmetric technology partnership, proposed a four-element model comprising capability, goodwill, self-reference, and behaviour of an individual or an organisation. Capability refers to future-oriented cooperation, necessary skills and technical knowledge to reflect the required learning dynamics, and a revival in technological partnership. Goodwill is understood as moral responsibility and good intentions. The behavioural component facilitates the transmission of intentions and vision of partnership through open communication, keeping promises, integrity, and commitment. Self-reference is, on the other hand, understood as individual or organisational identity containing clear values and culture, as well as mature perception of capabilities. It assures the awareness of complementing competences in a partnership. The author of the model indicates that there is a hierarchy within the presented components, wherein capability and self-reference comprise the necessary grounds for building a partnership, and the behavioural components and goodwill facilitate active expression of intentions. Literature also provides examples of one-dimensional conceptualisations of the interorganisational trust [8]. It is also worth mentioning that in the literature two methods of measuring trust may be encountered: the first, one-dimensional, is concentrated on the trust of one of the parties towards the partner [31], whereas the other focuses on mutual trust between the partners, or absence thereof [35]. Despite the fact that the role of trust in establishing, maintaining, and developing interorganisational relations is significant, often desired and favourable, overly high interorganisational trust may also cause some threats. They include, for instance, leaving the organisation vulnerable to opportunistic behaviours of the partner, limited monitoring of the environment and relying too much on information provided by the partner in the relation, and developing habits that may lead to passing over other collaboration alternatives that may be attractive for the organisation [36]. Therefore, in the practice of enterprise collaboration, due to the multi-dimensionality of their relations, the co-existence of trust and distrust is quite common, which is a peculiar paradox of interorganisational relations management.

## 3. Interorganisational distrust and its dimensions

Thus far, the literature has not focused much on the conceptual equivalent of trust, i.e. distrust. The researchers focused their attention mainly on how to develop and maintain or restore trust. Distrust was regarded as its opposition placed on the opposite end of the continuum, deeming high trust as equivalent to low distrust, and vice versa [37]. Having made such an assumption, there was no point in studying distrust. Only when distrust was deemed a separate construct did it result in a considerable growth in the number of publication on the topic [2, 38]. Currently, researchers indicate the need for broader research of distrust in interorganisational relations, pertaining, inter alia, to their sources and impact on business results [6, 8, 39].

In the literature, distrust is presented as a multi-dimensional concept covering rational and alleged mistrust. In the past, researchers ascribed to mistrust such qualities as: confrontationality, craftiness, hostility, lack of concern and respect for others, and egoistic and irresponsible behaviour [40]. Currently, it is regarded as a belief and expectation that the motives, intentions, and behaviours of the partner constitute a threat to one’s own interests. Distrust is defined most often as a belief that an agent will operate solely in their own interest, passing over the partner’s interests, despite any feeling of guilt [41], or even that, in order to guarantee one’s own interest, it may include involvement in behaviours that are detrimental to the partner [42].

Distrust and trust are related to action, and their emergence generates the need to make a decision regarding relations with the partner. However, contrary to trust, which encourages one to enter the relationship, distrust is related rather with withdrawal from the relationship and the need to safeguard against expected negative actions of partners.

It is worth noting that the literature more often draws attention to the fact that distrust is not a dangerous and destructive element of interorganisational relations, but a natural state typical for acute and prudent parties to the relations, who are aware of the mechanisms that drive people and their actions. In the event of different values and interests, combined with rather unsuccessful prior interactions, distrust may be a defensive response safeguarding against potential damage. On the other hand, trust combined with verification of the partner’s behaviours is a method of collecting information that may provide knowledge of potential abuse of trust or allow positive judgement of a partner who to date has not been trusted. As interorganisational relations are becoming increasingly complex and multi-faceted, they usually cover both trust and distrust, due to being satisfying in some aspects and disappointing in others. They are, by nature, ambivalent, as they are a combination of positive and negative states [43]. Moreover, levels of trust and distrust may change independently over the entire period of the relationship.

The achievements of researching distrust are much more modest that those of the studies of trust. Therefore, thus far, no satisfactory dimensions of distrust in analysed research contexts have been developed. In this case, in reference to distrust in interorganisational relations, it would be natural to assume dimensions analogous to trust, which would be consistent with the belief that both these constructs comprise the same elements, i.e. competence-based distrust, integrity-based distrust, benevolence-based distrust or goodwill-based distrust and competence-based distrust. In this context, the opposition dimensions of mistrust are pointed out such as incompetence, malevolence, and deceit [44]. Incompetence is defined as the belief that a partner is unable to meet his obligations due to a lack of knowledge, resources or responsibility, malevolence as a belief in the partner’s bad intentions and deceit the conviction of his dishonesty and willingness to cheat or use his partner to gain an advantage [44].

McKnight and Chervany [45], however, challenge the possibility of assuming identical dimensions of trust and distrust due to the fact that they are based on fundamentally different sets of emotions, which, in their opinion, allows the supposition that the relations between them are more orthogonal in nature. These authors proposed a model, worth quoting, which encompasses the dimensions of distrust in interpersonal relations, analogous to the trust model they presented. They isolated the following components of the model: disposition to distrust, institution-based distrust, distrusting beliefs, distrusting intentions, and distrust-related behaviour. These components remain within the scope of interest of other authors studying distrust also in the interorganisational context.

Similarly to trust, distrust is also described as a belief or as a behaviour. Distrust as a belief is a judgement or expectation of undesired behaviour of the partner resulting from acquired information regarding the partner’s intentions and actions. As opposed to lack of trust, which is most often related to lack of reliability or competence attributed to the partner, distrust pertains rather to beliefs regarding hidden motives or incompatible values subscribed to by the partner [46]. Distrusting beliefs–these express the degree to which the trustor is convinced that the trustee has no properties useful in a specific situation. In the context of interorganisational relations, this would be the belief of distrust of the trustor as the focal firm in relation to the trustee as a partner organisation or considered as a potential partner. In this context, four distrusting beliefs were distinguished: referring to (1) competence, i.e. belief that the partner does not have competence or power allowing them to be successful; (2) benevolence, i.e. belief regarding the unwillingness to act in favour of the partner’s interests; (3) integrity—belief that the partner does not act in good faith by not telling the truth or not fulfilling promises; and (4) predictability, i.e. belief that the partner’s actions are not consistent enough to be predictable in a specific context [44]. Behaviours expressing distrust include reduction of expenditures, withholding and twisting information, increasing control and monitoring, and establishing transaction collateral, including refraining from the transaction [47]. Disposition to distrust, on the other hand, means the degree to which a specific person demonstrates an unwillingness to rely on others due to attributed qualities of dishonesty, kindliness, competence, and predictability or the belief, regardless of the qualities attributed to individuals, that better results may be achieved assuming that people generally act in an unconsidered and unreliable manner. This dimension could be called distrust propensity, because it seems analogous to trust propensity, which is well described in the literature [48]. In the presented model [45] also distinguish distrusting intentions related to belief and an unwillingness to rely on another party, regardless of the consequences. This dimension, however, seems to be somewhat coincident with disposition to distrust; in particular, is it is considered in the context of interorganisational relations.

Institution-based distrust means a belief that there are no institutional conditions assuring the success of an enterprise, expressed in the absence of structural assurance, i.e. a belief that the formal structures (warranties, regulations, promises, legal provisions, and other procedures) are aimed at promotion of the achievement of goals and the absence of situational normality, which, in turn, means a lack of faith that the organisation’s environment is properly organised, beneficial, and favourable to achieving success. A third dimension is more often proposed in the literature, pertaining to the consistency of values and norms [49]. The dimensions presented above seem to be useful for the study of interorganisational distrust. They include both the determinants of distrust that are most likely to have an impact on decisions, which may be then shared by a wider group of decision-makers; this stresses the significance of distrust to a partner institution which is often based on its operational standards, organisational culture, or opinions of a third party and indicates the importance of beliefs/experience of cooperation that affect the forming of distrusting beliefs.

As we pointed out earlier, little research has so far been devoted to distrust in inter-organisational relationships. At the same time, it is worth noting that more and more research is currently being conducted in this area. However, most of the studies on this issue are conceptual [2, 3]. A significant number of studies use a qualitative or experimental approach to research [4, 5]. Table 1 presents the scales used in the B2C and B2B studies.

**Table 1:** Scales for studying distrust in relationships.

Author	Country	Sample	Relationship type	Number of items/dimensions	Comment
Cho [8]	not mentioned	on line buyers of books and clouds	buyer-seller	4	distrust treated unilaterally negatively, one-dimensional construct, scale relates to B2C relations
McKnight, Choudhury, [50]	USA	students from a computer literacy course	buyer-seller	Two dimensions: distrusting beliefs (11) and distrusting intention (5)	scale relates to B2C relations
Şengün et al., [51]	Turkey	drug industry	small business dyad (buyer supplier)	6	reference to a narrow type of relationship, distrust identified only with opportunistic behaviour, one-dimensional construct
Raza-Ullah, Kostis, [24]	Sweden	high technology manufacturing and knowledge- intensive services industries	coopetition	3	distrust treated as one-dimensional construct

The Table 1 presents the few scales used by the authors to study distrust in different types of relationships, which formed the basis for creating our scale. Also included are scales used to study distrust in B2C e-commerce. It is worth noting that the authors of these scales mainly refer to conceptual articles on distrust in the process of scale development. Therefore, there is a need to develop a scale to study distrust in interorganisational relationships.

## 4. Relations between trust and distrust

The question of whether trust and distrust are opposing phenomena in a single dimension or whether their relations are more complex has drawn the attention of researchers [52, 53]. Currently, a growing number of researchers are leaning towards the opinion that trust and distrust are differing and separate constructs but are related to each other [6, 54]. This means that lack of trust does not have to be related to high distrust, and low distrust to high trust. However, the connection of these two constructs assumes that, to a certain degree, they have an impact on each other, e.g. a drop in distrust may have positive consequences for trust, and, on the other hand, a high level of distrust will probably be a symptom of low trust in the relationship.

Historically, the original views regarding the relations between trust and distrust treated both concepts as opposing states located at two opposite ends of a single continuum [52]. The researchers of this stream, however, are not in agreement as to how trust transforms into distrust and vice versa. Some of them believe that there is a field reflecting the neutral state in which individuals are neither highly trusting nor highly distrusting [e.g. 55]. The researchers leaning towards this view notice that loss of trust is not always connected with the development of distrust [56]. They stress that distrust may be reduced without inducing trust and vice versa [55]. This approach suggests, to a certain extent, that trust and distrust are separate constructs [6]. On the other hand, if one was to assume that trust and distrust are opposites located on one continuum, there would be no point in treating them as separate constructs [53].

Current research shows that various behaviours of the partner to the relation may have an impact on the level of trust or distrust (i.e. increase or reduce it), e.g. one may trust the competences of the other party and experience distrust to the partner’s reliability [43]. In addition, in some relationships, the co-existence of, for instance, distrust towards the kindliness of the partner and trust in their competence may allow the relationship, although not fully satisfying, to achieve the assumed goals of the parties. In another case, the same combination of trust and distrust may result in discontinuing the relationship.

**Fig 1:**
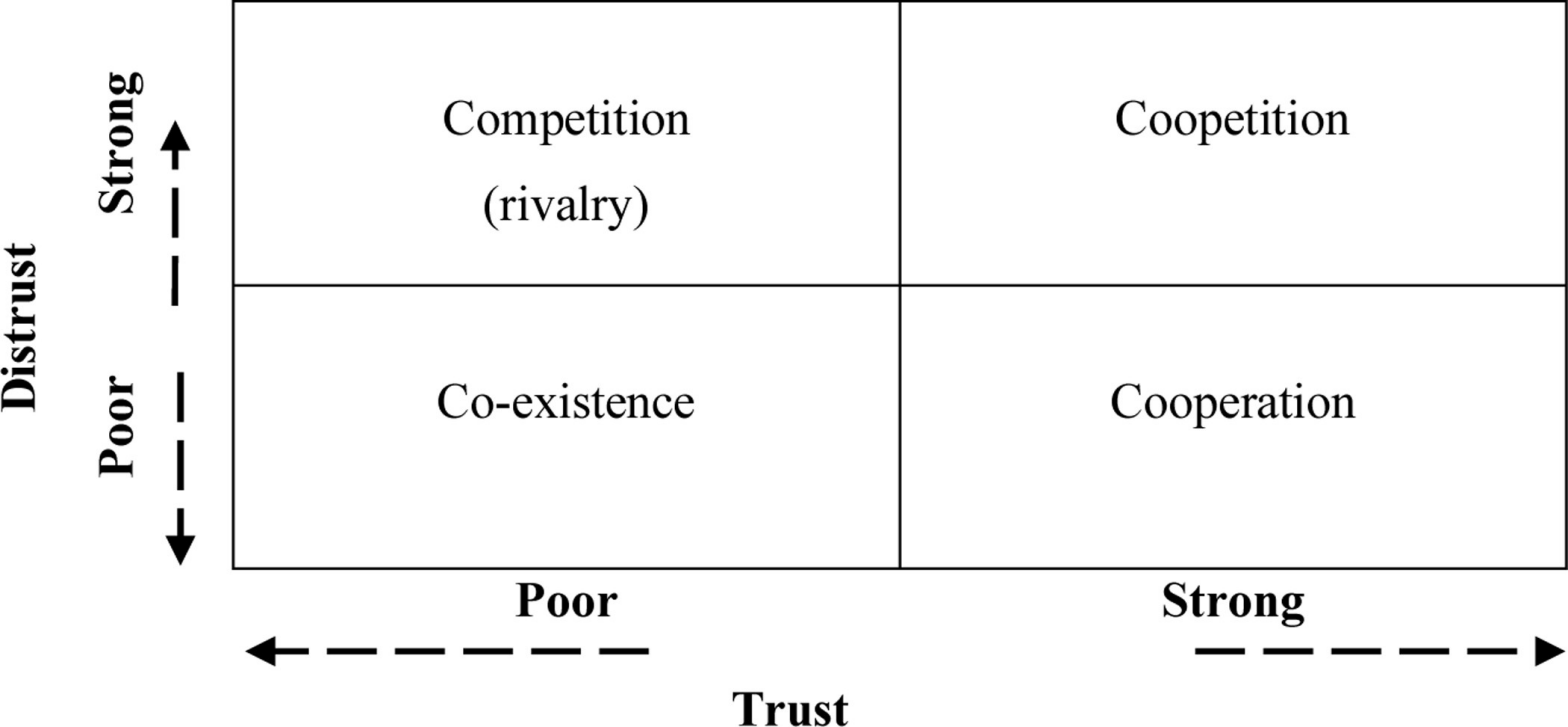
Relations between trust and distrust by nature of interorganisational relations.

McKnight and Chervany [45] claim that a high level of trust should be accompanied by some distrust, which helps identify potential problems and solve them relatively quickly without exposing the partners to the loss of trust. The above deliberations lead to the conclusion that relationships of any type have a complex and multi-dimensional structure by combining both trust in and distrust towards partners, which may be manifested in concurrent collaboration and competition in a given relation. Therefore, it is probable that the level of trust and distrust between entities may be a factor that describes the nature of the relationship between them, which is presented in Fig 1.

The analysis of the above examples leads, first of all, to the conclusion that there is some level of distrust in the relations of cooperation. A similar relation applies to coopetition, which assumes simultaneous cooperation and competition. It seems that coopetitive relations assume trust within the framework of jointly achieved goals and projects, but also a certain level of distrust in the area of actions carried out outside the established partnership. One may expect also that having a coopetitive relationship with a partner wherein trust will be maintained may result in limited inclination for opportunistic behaviours. The competitive relation, on the other hand, is characterised by high distrust and low trust, which means that past experience had formed and established an attitude of active distrust and lack of trust between entities. A high level of active distrust generates the need for constant monitoring of the competitor’s actions and carrying out pre-emptive measures to guarantee the achievement of one’s own goals. On the other hand, the co-existence relations are characterised by a low level of distrust and trust, which may be related to the low level of co-dependence or lack of sufficient knowledge and experience related to the given entity that would justify active trust or distrust. It should then be concluded that trust and distrust are simultaneously present in all types of interorganisational relations.

Therefore, apart from the epistemological, the study of the relations between trust and distrust also has some practical value. The identification of mutual relations between these constructs is important from the point of view of answering e.g. the question of whether actions aimed at the development of a relation hindered by high distrust may be supported by building trust. In other words, the identification of the nature of this relationship should provide comprehensive clues regarding the management of trust and distrust in relations.

## 5. Method

The research conducted with full attention to ethics clearance. Participants of research informed about the purpose of the research and its anonymous character. Participants may resign from the project at any time during the project. Respondents was also notified about the manner in which the data will be an analyses and their potential access to the research results.

### 5.1. Item development process

We conducted a search for journal articles within the Science Direct, Elsevier, Emerald, and EBSCO information sites using key words such as “interorganisational trust”, “inter-organizational trust” and “trust between the organizations”, “interorganisational distrust”, “inter-organizational distrust” and “distrust between the organizations”. We then analysed the results in order to find research of relevance to our study. Thus, at this stage studies dealing with intraorganisational trust or trust within an organisation were left aside. Deductive method was used to identify preliminary items for the scales. Based on literature review, the researchers developed a collection of propositions allowing the definition of the trust and distrust in relations with partners. In the case of trust, we took into account the two dimensions of competence and good will [20, 34]. In the case of distrust, the dimensions of distrusting beliefs and distrusting intention were taken into account [50].

### 5.2. Expert research

Expert research was conducted **from May to June 2018**. The purpose was, on the one hand, to learn the opinion of experts regarding the relational strategy in the context of strategic choices made, as well as its qualities, and on the other hand, the acquisition of notes, comments, and suggestions of experts regarding the developed tool for studying relational strategies of enterprises, concerning also scales for researching trust and distrust. **Twenty experts**, representatives of the **Polish academic community**, were invited to participate in the research. They were selected on purpose, and the selection criteria were as follows: considerable achievements in strategic management and/or topics related to interorganisational relations of networks, which was verified based on the number of publications and the topics thereof on the basis of google scholar data base, obtained the academic post-doctoral degree, and had experience in academic research. Nowadays, it is indicated that when conducting expert research, one should primarily take care of proper selection of experts, who may have an individual and qualitatively valuable contribution from the perspective of the research objectives, whereas their number is of no greater importance [57, p. 309]. In light of these opinions, it may be concluded that the group of 20 experts selected according to the presented criteria was sufficient and justified in the context of the research goal. The expert questionnaire was sent to experts by electronic and traditional mail. Each expert was asked to **express their opinion regarding statements describing individual latent variables** studied from the perspective of their legitimacy and theoretical validity, as well as the degree of understanding by economic practitioners. As regards individual statements, the experts expressed their opinions indicating one of three possible answers, namely: **yes** (keep)–the statement measures the specific latent variable and is correctly formulated, **no** (reject)–the statement does not measure the specific latent variable or is redundant in the context of other statements, **modify**–the statement is required for the measurement of latent variable but requires reformulation to make it more adequate and clear. If this option was selected, the expert was asked for a proposal of such reformulation. Based on the obtained results, the originally proposed scale was modified.

### 5.3. Data collection

In the next stage, empirical research was conducted from January until the end of May 2019, involving a group of 400 entities operating in Poland. The sample **is representative** in terms of employment size and Polish Classification of Activity. In this group, there are entities with qualities ascribed to small companies (i.e. with 10–49 employees), medium-sized companies (i.e. with 50–249 employees), as well as large companies (i.e. with over 250 employees). The sampling frame was the database of the National Business Register (REGON), and the research was carried out using the method of probabilistic layered random selection. The survey was conducted using a CATI questionnaire (Computer Assisted Telephone Interview). The questionnaire was a structured and standardised. Respondents were either owners or top management. The studied population were mainly mature entities, i.e. operating on the market for over 20 years. They therefore had appropriate experience in creating, developing, or withdrawing from interorganisational relations. Trust and distrust were among the partial issues.

### 5.4. Measurement of variables

Trust and distrust were measured according to the level and nature. **The levels of trust and distrust** were determined by the respondents in relation to the selected groups of partners, i.e. suppliers, customers, competitors, and other key entities with which the enterprise competes. They made an assessment using a seven-point Likert scale with 1 corresponding to definite lack of trust and 7 to definitely high trust. On the other hand, the **nature of trust** was defined using eight items, and the **nature of distrust** by six items, with regard to which the respondents expressed their opinions using a seven-point Likert scale, from 1—strongly disagree to 7—strongly agree. PS IMAGO PRO 6.0 was used to analyse the results.

To reduce common method bias we followed several techniques that Podsakoff [58] suggest. For procedural remedies, we took several approaches, including 1) using multiple-item constructs to capture all of the key variables and grouping the construct items in sections instead of variables, 2) assuring respondents that there were no right or wrong answers and encouraging them to respond as honestly as possible, 3) avoiding ambiguous questions and vague concepts and constructed items carefully.

Moreover, additional tests are carried out to avoid some biases: first, condition indexes are below 30 and VIF values are below 3.3, (1.39; 1.07) suggesting an absence of significant multicollinearity between independent variables [59]; secondly, a single Harman factor test is performer–to see the total variance extracted once we force all our items to be grouped into a single factor, and this variance is not exceeded of 50%.

## 6. Results

### 6.1. Results of expert research

The conducted statistical analysis in the case of testing both scales comprised the following stages: analysis of variable values distribution, building a matrix of correlation between original variables and initial analysis thereof, exploratory factor analysis, confirmatory factor analysis, and, in the case of the distrust variable, an attempt to build a model of the relationship between construct components.

Following the review of literature, a collection of statements was developed to study the mechanisms of trust. They are presented in Table 2 along with adequate references and accuracy assessment of the measurement of trust-building mechanisms in the experts’ opinions.

**Table 2:** Operationalisation of the construct of mechanisms of building trust in partners as a latent variable.

Operational measurement of construct	References	Experts’ opinion (N)		
		Keep	Reject	Modify
1. We expect that we will gain benefits exceeding the costs of cooperation	Shapiro [60]; Ganesan [61]; Chow & Holden [62]; Doney & Cannon [63]; Raza-Ullah, Kostis [24]; Mc Knight & Chervanny [45]; Pavlou et al. [64]; Lui & Ngo [65]; Jiang et al. [66]; Czakon & Czernek [67]	12	5	3
2. We assess that, based on their competence, the partner may meet the terms of cooperation		14	4	2
3. We perceive the partner as predictable		14	3	3
4. We perceive the partner as honest/acting based on specific principles		14	3	3
5. We assume that our partner also strives for cooperation		13	4	3
6. Partner’s reputation allows a positive assessment of their credibility		15	3	2
7. We trust the partner we do not know if another entity (business, organisation, authority) we trust regards them as credible		15	4	1

In the case of the construct of building trust in partners, most experts agreed with the proposed statements; however, some experts rejected them. An argument presented by experts who expressed a negative opinion was primarily that the proposed statements did not refer to mechanisms but rather presented “*reasons*, *grounds* [for trusts to partners]”, “*describe feelings regarding partners*”, study *attitudes to and expectations regarding the partners*”. One of the experts concluded that if “*trust is a construct comprising calculation*, *circumstances*, *risk*, *principles*, *intentions*, *feelings*”, then the proposed operationalisation is adequate, and another expert was of the opinion that “*since it comes to ‘mechanisms’ and not a specific mechanism*, *the match specific individual scales to a general construct cannot be assessed*”.

Some experts suggest the modification of individual statements by making them more specific, and thus:

- as regards statement 1, one expert proposed the determination of what kind of benefits are discussed (where they come from), another expressed the opinion that “*trust cannot be built on mere expectation of benefits exceeding cooperation costs; it is the foundation of calculated involvement*”, and another expert proposed that it should be complemented with the phrase “(…) *as a result of mutual trust we have built*”. A fourth expert believed that it did not fit the construct at all;
- as for statement 2, one expert proposed that the phrase “*meet the terms of cooperation*” be replaced with “*fulfil goals of cooperation*”;
- as for statements 3, 4, and 5, one expert was of the opinion that the kind of partners that are assessed should be determined; whereas, as regards statement 5, another expert added that “*since we are cooperating with a given partner*, *we rather assume that they strive to achieve jointly established goals of relation*” and therefore proposed the following reformulation: “*we assume that our partner is also striving to achieve jointly established goals of relation*”;
- regarding statement 6, it is proposed that only the phrase “*to assess*” is accepted, without the adverb “*positively*”, and that statement 7 should be modified as follows: “*We start cooperation when another entity (business*, *organisation*, *authority) we trust regards the other entity as credible”*.

Moreover, one expert suggests that, in relation to all statements, the following questions should be answered accurately: “*Who is being assessed*, *a potential or the current partner in cooperation*, *and is that supposed to be a term of cooperation*?”.

When evaluating the entire construct, 12 people regarded its operationalisation as satisfactory, three people deemed it wrong due to it being determined from the angle of mechanisms, whereas five proposed that it should be complemented by the aforementioned clarifications and issues such as: “*communication (knowledge and vital information sharing procedures*, *conflict solving mechanisms*, *etc*.*)*” or the fact that “*relations with trusted partners are extended*”. Finally, one of the experts stressed that the structure of the construct contains items that refer to calculational as well as relational trust and added that “(…) *it is worth remembering that*, *in many studies*, *both perspectives of trust are regarded as two separate variables*. *Perhaps it is worth paying more attention to those two separate mechanisms–ex ante trust (calculational) and ex post trust (relational) etc*.*–and building a measurement tool in such a way as to conclude which mechanism is of more importance*”.

Taking the expert’s remarks into account, the name of latent variable was changed from “*mechanisms of building trust in partners*” to “*trust in partner in the relation*” by adequately reformulating the individual statements. Moreover, an additional statement was added which allowed the determination of the level of trust in key partners within the scope of basic groups of entities in the value network, i.e. suppliers, customers, competitors, and stakeholders (i.e. other vital cooperating entities). Table 3 presents the modified operationalisation of this construct.

**Table 3:** Operationalisation of the trust in partners in relation construct—after modification proposed by experts.

Describing statements	Evaluation by managers
1. We trust our key partners, including:	Managers are given the task to assess the individual statements using the **1–7 scale**, where 1 means strongly disagree and 7 means strongly agree
suppliers	
customers	
competitors	
other key entities we cooperate with	
2. We trust that the cooperation will yield benefits exceeding costs	
3. We trust that, based on their competence, the partner may meet the terms of cooperation	
4. We trust that our partner is striving to achieve shared goals	
5. We trust that in the event of any dispute, we will find a solution together (with the partner)	
6. We perceive our key partners as predictable/acting based on specific principles.	
7. We perceive our key partners as honest	
8. When an entity (business, institution) we trust regards another entity as credible, we also trust them in the event of potential cooperation	
9. Our to-date experience with key partners is positive	

A similar procedure has been applied in reference to the operationalisation of the construct of distrust of partners; the describing statements with adequate references and expert opinions regarding the accuracy of statements are presented in Table 4.

**Table 4:** Operationalisation of the construct of distrust of partners as latent variables.

Operational measurement of construct	References	Experts’ opinion (N)		
		Keep	Reject	Modify
1. Regardless of the partner’s reputation, each cooperation is burdened with considerable risk	Lewicki et al. [12]; Grover & Malhotra [68]; Cho [6]; McKnight & Choudhury [50]; Sengun & Wasti [51]; Dimoka [54]; Moody et al. [44]; Moody et al. [69]; Raza-Ullah, Kostis [24]	18	2	0
2. We are sceptical about cooperation with a new partner		17	2	1
3. Successful cooperation with a partner in the past does not guarantee the same in new undertakings		18	1	1
4. The fact that another entity regards a partner as loyal does not mean that this partner will be loyal to our company		18	1	1
5. Competences of a partner are not their constant feature. Considering the volatility of environment and conditions in organisations, it can be expected that a partner will not be able to act equally competently in any situation		17	1	2
6. One cannot be certain that even a proven partner will in each case try to meet the terms of cooperation with equal honesty		16	2	2
7. Despite prior arrangements and agreements, I believe that partners should be controlled (in the cooperation process)		19	1	0

A great majority of experts agreed with the proposed statements describing distrust towards partners. One of the experts rejected them all concluding that “*these categories do not constitute a scale; wouldn’t it be simpler to ask to what extent we trust or distrust a partner on the scale from 1 to 10*?”. Because this opinion is a shared one, additional statement was added (similarly as in the case of trust), referring to the level of distrust towards key partners in individual groups that make up a value network. Moreover, one expert suggested that three statements, namely 1, 2, and 6, should be removed from the proposed catalogue, and several experts proposed slight stylistic modifications of some statements. What is more, as regards statements 6 and 3, one of the experts noticed that they carried the same information.

Consequently, except for two experts, the others assess the structure of the construct as satisfactory, and some of them reported certain remarks. In the view of new expert, it is worth reformulating individual statements “*pejoratively (*…*) and not with double negative*”. Another expert indicated that there was a “*high probability that everyone will respond* [to statements formulated as such]—*strongly agree*”, which may cause difficulties later in the interpretation thereof. Yet another expert reported a remark as regards the “*sense of measuring DIS-TRUST*” and still another expressed the opinion that it “*all depended on the response to question in the previous case regarding what trust was*”.

While being in a position that distrust accompanies trust in interorganisational relations, and in order to determine the level of distrust towards key partners and attitudes that accompany it, one statement was added to the construct and the others were modified to allow the best identification. Table 5 presents the operationalisation of distrust towards partners after considering the experts’ remarks.

**Table 5:** Operationalisation of distrust towards partners in relationship construct—after modification proposed by experts.

Describing statements	Evaluation by managers
1. We express some distrust towards our key partners, including:	Managers are given the task to assess the individual statements using the **1–7 scale**, where 1 means strongly disagree and 7 means strongly agree
suppliers	
customers	
competitors	
other key entities we cooperate with	
2. We are sceptical about cooperation with a new partner	
3. Despite the fact that the partner has the necessary competences, they cannot fully use them at each stage of cooperation	
4. Successful cooperation with a partner in the past will guarantee the same in new undertakings	
5. A proven partner will in each case try to meet the terms of cooperation with equal honesty	
6. Despite prior arrangements and agreements, partners should always be controlled in the cooperation process	
7. The fact that another entity regards a partner as credible means that this partner will be equally honest to our company	

The conducted expert research increased the theoretical validity of the developed tool to study trust and distrust in interorganisational relations.

### 6.2. Results of statistical analyses

The conducted statistical analysis in the case of testing both scales comprised the following stages: analysis of variable values distribution, building a matrix of correlation between original variables and initial analysis thereof, exploratory factor analysis, confirmatory factor analysis, and, in the case of the distrust variable, an attempt to build a model of the relationship between construct components.

#### 6.2.1. Trust scale

Firstly, the distributions of variable values in the sample were studied. The achieved results, were focused mainly on the positive side of the scale. The in-depth analyses demonstrated that the dispersion of responses was higher in small enterprises. Lower scores were more frequent than in the case of medium-sized and large enterprises. The larger the enterprise, the higher the likelihood of moderately positive responses to questions regarding trust.

In the next step, the correlation between items was studies, involving the *Pearson’s* r *(correlation coefficient)*, which is presented in Table 6.

**Table 6:** Matrix of correlation between variables on trust.

	T1	T2	T3	T4	T5	T6	T7	T8
T1								
T2	.934**							
T3	.965**	.961**						
T4	.941**	.918**	.950**					
T5	.958**	.950**	.979**	.937**				
T6	.956**	.956**	.978**	.941**	.977**			
T7	.843**	.791**	.825**	.836**	.816**	.833**		
T8	.772**	.782**	.804**	.751**	.811**	.822**	.675**	

** Correlation is significant at 0.01 (two-sided).

The analysis of obtained results gave grounds to assume a factor model as an accurate technique for the analysis of collected data. The correlations between variables proved to be strong and statistically significant. Therefore, all variables could be used to build interorganisational trust scale. Only items T7 and T8 demonstrated slightly poorer relations with other ratios. In the next step, exploratory, principal-component factor analysis (PCA) was conducted to check whether the items defining the nature of trust comprise one or a number of factors, and to reduce items that did not load on the appropriate component of the dimensions of interorganisational trust. The Jolliffe criterion was selected as a criterion for isolation of components (component eigenvalue over 0.7). The analysis of main components indicated, however, a one-dimensional solution. The Kaiser-Meyer-Olkin (KMO) statistic reached 0.959, which is high and corroborates the legitimacy of factor analysis. Table 7 shows the obtained results.

**Table 7:** Eigenvalues, variance explained by solution with 1 trust component.

Component	Initial eigenvalues			Sums of squared loadings after isolation		
	Total	% of variance	accumulated %	Total	% of variance	accumulated %
T1	7.187	89.839	89.839	7.187	89.839	89.839
T2	.344	4.304	94.143			
T3	.252	3.149	97.292			
T4	.077	.965	98.257			
T5	.058	.724	98.981			
T6	.042	.529	99.510			
T7	.021	.265	99.775			
T8	.018	.225	100.000			

The dominating main component explained almost 90% of variances of the eight variables used in the analysis. The importance of other components is minimum. All variables used in the analysis are strongly related with the isolated dimension.

### 6.3. Construct reliability and validity

#### 6.3.1. Reliability

To assess the reliability of scale the following tools were used: path coefficients of individual items and squared multiple correlations (R2) as well as Cronbach’s alpha for assessing the reliability of latent component. The reliability statistics for the trust scale are shown in Table 8.

**Table 8:** The reliability statistics for the trust scale (obtained from confirmatory factor analysis).

Items	R 2	Loading	t-value	CR	AVE	Cronbach’s alpha
T1	.944	.972	a	0.984116	0.886148	.983
T2	.932	.966	54,920*			
T3	.984	.992	72,759*			
T4	.912	.955	50,592*			
T5	.974	.987	67,914*			
T6	.976	.988	68,772*			
T7	.702	.838	28,670*			
T8	.663	.814	26,466*			

Notes: The t-value is not available because the coefficient is fixed at 1

*p-value<0.001, **p-value<0.05 chi-square = 127,232; df = 20; chi-square/df = 6.362, p = 0.00; GFI = 0.921; AGFI = 0.857; RMSEA = 0.097; NFI = 0.981; CFI = 0.984 =; AIC = 159,232

All the items were significantly related to interorganisational trust construct, verifying the posited relationships among the indicators and constructs. The squared multiple correlations (R2) were over the limit of 0.5 [70]. All loadings of the items turned out to be very high, and that-values associated with each of the loadings exceeded the critical values for the 0.01 significance level, implying good reliability on the item level. Cronbach’s alpha, similarly to CR, reached a high value of 0.948, exceeding the recommended value of 0.94 [71]. This value may suggest that the respondents did not fully distinguish the meaning of individual items.

#### 6.3.2. Construct validity

Convergent validity is related to how strictly the new scale is linked to other variables and other measures of the same construct [72]. Strong evidence is achieved when the squared factor loading (R2) is greater than 0.5. Factor loadings for all of the items of the scale were greater than 0.6, and all were statistically significant at the 0.01 significance level. These variables are strongly correlated with the latent variable at a similar level. The AVE value reaches a satisfactory value of 0.87, thus evenly distributing the variance among all items.

### 6.4. Discussion regarding the scale of interorganisational trust

The present research attempts to test a multidimensional solution by describing a step-by-step approach to developing the construct of interorganisational trust and a valid measurement scale for assessing employee perception of impersonal organisational trust. To this purpose, an expert method was used to construct the questionnaire, based on which the individual items were modified and refined. Unfortunately, the approach adopted did not reveal the internal structure of intra-organizational trust. As a result of the conducted research procedure, a one-dimensional solution was developed. A single scale is composed of all eight variables. The additional analysis of the sample with division by enterprise size also did not demonstrate a more complex structure of interorganisational trust.

A frequent approach in the study of trust in relation to different entities/objects are attempts to copy the three-element ABI structure (ability, benevolence, integrity) extracted in the study of interpersonal relations and adapt it to the characteristics of a specific designator. Such an attempt was also made in the case of inter-organisational trust, with benevolence and integrity being replaced by the joint construct of good will [20]. It should be emphasised that the previous research on trust, although it concerned inter-organisational relations, was often conducted in the interpersonal dimension, e.g. on relationships in buyer-seller, supplier-manufacturer [12, 31]. Few studies have concerned interorganisational relations understood as collective mental state and collective decision to rely on the trustor as the focal firm and the trustee as a partner organisation [20, 66].

Thus, on the basis of the obtained results it may be presumed that in the case of treating trust in relationships as a generalised construct separated from the specific interpersonal relationship between partners the sub-constructs characteristic of interpersonal trust or their derivatives do not occur. Finally, it is worth pointing out the conclusions for the use of the questionnaire resulting from the statistical analysis carried out. In order to shorten the questionnaire, use of a few statements instead of the entire scale may be recommended. Paradoxically, in the case of such strongly correlated and homogeneous variables, the recommended solution would be to use variables that are slightly less well correlated with the scale and differing in terms of descriptive statistics and distributions (e.g. four variables, including T7 and T8).

Again, it is worth drawing attention to the high concentration of responses in moderately positive categories. Responses in small enterprises are more dispersed than in medium-sized enterprises, in particular in comparison to large ones. This may be connected with the selection of sample, which included only mature entities, i.e. operating on the market for over 20 years. One may assume then that the broad experience of these enterprises related to the creation, development, and withdrawal from relations allows the manager to notice, appreciate, and use the value of trust in interorganisational relations.

#### 6.4.1. Distrust scale

In the case of the distrust scale in interorganisational relations, a significantly higher diversification of responses was noted. Respondents used all items of the scale (although rarely from extreme positions). Questions DT2, DT3a, DT4a, and DT6a (invereted) were characterised by lower average values than the remaining statements, whereas the respondents evaluated items DT1 and DT5 in a quite extreme manner (low percentage of responses in a neutral position). DT5 concentrated responses in two categories: 5 and 6.

In the next step, the correlation between items was studies, involving the *Pearson’s* r *(correlation coefficient)*, which is presented in Table 9.

**Table 9:** Matrix of correlation between variables on distrust.

	DT1	DT2	DT3a	DT4a	DT5	DT6a
DT1						
DT2	.449**					
D3a	.213**	.215**				
DT4a	.164**	.244**	.880**			
DT5	.277**	.034	.099**	.110**		
DT6a	.138**	.005	.515**	.470**	.137**	

** Correlation is significant at 0.01 (two-sided). * Correlation is significant at 0.05 (two-sided)

Variables DT3a, DT4a, and, to a lesser extent, DT6a correlated with each other, and concurrently, although to a slight extent, connected with other variables. Variables DT1 and DT2 form their own system, whereas UT5 demonstrated very poor correlation with other variables (although high enough for the dependence to be statistically significant).

The initial analysis of main components conducted on the full set of six variables resulted in the isolation of three dimensions (using the Kaiser criterion—eigenvalues of dimensions higher than 1), which explained almost 81% of variances of used variables. The KMO value was 0.589, which is acceptable but quite low. However, the in-depth analysis revealed some problems with such a solution. Most of all, the DT5 variable, poorly correlated with other variables, created its own dimension (the DT1 variable was also correlated with this dimension), which was poorly correlated with the first (0.113) and second dimension (0.104). In addition, the inverse image matrix used for diagnostic purposes allowed us to notice the unacceptable KMO value of the variable (0.433). KMO values for variables DT1 and DT2 were slightly higher but also below 0.5. Therefore, it was decided to remove the DT5 variable from the analysis, which yielded the desired effect. KMO values for DT1 and DT2 over the threshold value of 0.5 increased. Two factors explained 75% of the variability of the applied variables.

The two dimensions obtained from the conducted analysis are presented in Table 10. The presented factors are correlated with each other at 0.243. In order to discover the correlation between dimensions, the PROMAX rotation was used once more (kappa = 4).

**Table 10:** Two factors obtained in a variable distrust.

Items	Factor 1	Factor 2
UT3a	.916	
UT4a	.899	
UT6a	.783	
UT2		.873
UT1		.828

The assessment of factor loadings in the model matrix allows us to conclude that the first dimension is built by variables DT3a, DT4a, and DT6a, and the second one by variables DT1 and DT2. Variables are strongly correlated with the dimension to which they were assigned. As a result of the conducted research, the two following distrust levels were isolated: disposition to distrust (variables DT1 and DT2) and distrustful beliefs (variables DT3a, DT4a, and DT6a).

### 6.5. Construct reliability and validity

#### 6.5.1. Reliability

Due to the fact that the second factor comprises only two items, only the first factor was subject to reliability analysis using Cronbach’s alpha. It is assumed that the latent variable should be made up of three items; therefore, the value of Cronbach’s alpha of 0.62 for the second factor is only informative in nature. Table 11 shows the correlation coefficients between the first factor and the variables that compose it.

**Table 11:** Correlation coefficients between the first factor of distrust and the variables that build it.

Items	Correlation coefficients
UT3a	.939
UT4a	.924
UT6a	.726

As can be noticed, the DT6a variable is more poorly correlated with the factor than variables DT3a and DT4a. Based on standardised position for scale built on the grounds of three variables, Cronbach’s alpha totalled 0.823, which is a high result. To check how a specific variable is related to the scale, an analysis of position statistics analysis as specified in Table 12 was carried out.

**Table 12:** Distrust scale items statistics.

Items	*x*¯	SD	Scale average after removal of position	Scale variance after removal of position	Total correlation of positions	Square of multiple correlation	Cronbach’s alpha after removal of position
UT3a	*3*.*05*	1.050	6.62	3.148	.795	.788	.634
UT4a	3.12	.948	6.55	3.566	.768	.775	.679
UT6a	3.50	1.119	6.17	3.752	.509	.266	.934

The removal of the DT3a and DT4a variables deteriorates the scale built based on the first main factor. It would be possible to remove the UT6a variable; however, the scale would be too short then (only two items). Therefore, it was decided to leave three variables. In the next step, the confirmatory factor analysis was conducted, which confirmed the to-date conclusions. The results of the confirmation analysis are presented in Table 13.

**Table 13:** The reliability statistics for the distrust scale (obtained from confirmatory factor analysis).

Items	R 2	Loading	t-value	CR	AVE	Cronbach’s alpha
Disposition to distrust				.857896	.68117	.823
UT3invert	.964	.982	a			
UT4invert	.804	.897	19,755*			
UT6invert	.275	.524	10,954*			
Distrust beliefs						
UT1						
UT2						

Notes: The t-value is not available because the coefficient is fixed at 1

*p-value <0.001, **p-value <0.05 (model parameters for two-element variables cannot be estimated [73]

**Fig 2:**
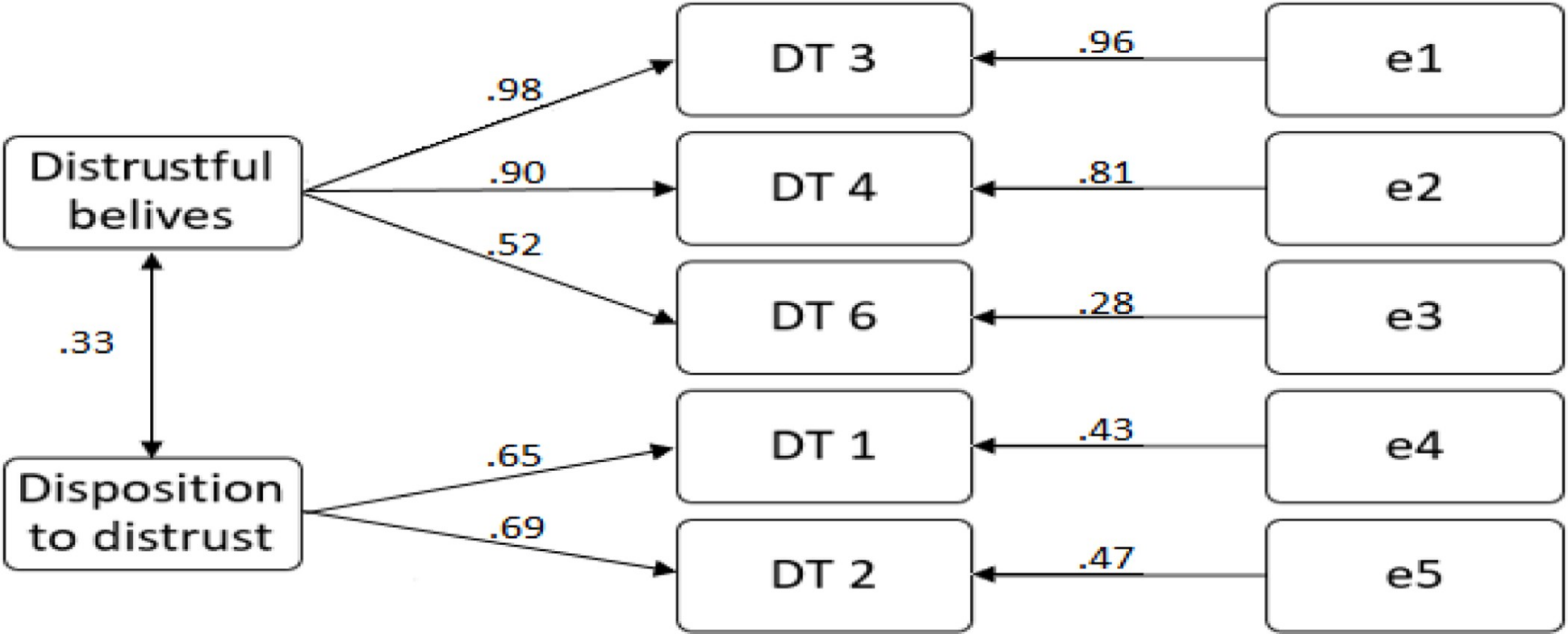
SEM model presenting relation between constructs of distrust. Chi-square = 23,512; df = 4; chi-square/df = 5876, p = 0.00; GFI = 0.978; AGFI = 0.916; RMSEA = 0.07; NFI = 0.973; CFI = 0.977; AIC = 45,515.

The given parameters demonstrated the need to reject question UT6a and keep the construct made up of two items. In order to determine the preferred model, SEM models were built for variants with two and three positions of the distrust variables, which showed that the model with two positions has better fit parameters. Therefore, it was decided that item DT6 should remain in the model. The correlation between S1 and S2 is statistically significant and totals 0.33. (Rejected model parameters: chi-square = 9385; df = 1; chi-square/df = 9385 p = 0.02; GFI = 0.989; AGFI = 0.885; RMSEA = 0.09; NFI = 0.987; CFI = 0.988 =; AIC = 27,385. This relationship is shown in Fig 2.

#### 6.5.2. Validity

*6*.*5*.*2*.*1*. *Convergent validity*. The factor matrix shows that variables are correlated with main factor (latent variable) at significant level (tab. 10). The AVE value reaches a satisfactory value of 0.68, thus evenly distributing the variance among all items.

*6*.*5*.*2*.*2*. *Discriminant validity*. Assessment of discriminant validity requires examination of the factors to ensure that they are not perfectly correlated, i.e. correlations equal 1 [74]. The main diagonal of Table 14 presents the square root of AVE, the value of which should exceed the correlation between variables.

**Table 14:** Variables intercorrelations between distrust components.

Variables	Distrustful beliefs	Distrustful beliefs
Distrustful beliefs	.825	
Disposition to distrust	-.330	.670

The obtained results show that the square root of AVE reached the required value.

### 6.6. Discussion regarding the scale of interorganisational distrust

The analysis of the distrust scale yielded a two-dimensional solution. The scale is made up of two variables.

- The **distrustful beliefs (DB)** variable comprises beliefs and elements of behaviours related to the distrust towards partners, which stem from the to-date experience with cooperation with these partners (items DT3a, DT4a, and DT6a). It covers such elements as the need to control partners despite prior arrangements or agreements, belief that a third party’s recommendation regarding the partner is unreliable, or even conviction that the joint positive experience from cooperation will be reliable in terms of new undertakings.
- The **disposition to distrust (DtD)** variable covers items that characterise the subjective or collective beliefs of respondents as regards the validity of distrust towards them, i.e. generalised distrust and belief that even if the partner has the competence, they may not use it in the cooperation process (items DT1, DT2).

The DT5 variable was excluded because it was poorly related to other items and did not diversify the responses which proved its uselessness to build the scale. Variables measuring distrust diversify the respondents quite well. They are highly correlated, and Cronbach’s alpha for the first factor reached a satisfactory result of 0.823. However, it is worth continuing the research of the scale with a view to studying the possibilities of using a larger number of items. Three items per dimension are regarded as a threshold value. Therefore, it is problematic to use dimensions built of two variables even in further confirmatory analysis.

### 6.7. Relations between trust and distrust

The next step is the analysis of the dependence between the researched constructs: trust and distrust. To that end, the correlation between variables was studied using Pearson’s linear correlation coefficient. This dependence proved to be in inverse proportion, which means that if the value of one construct grows, the value of the other decreases, whereas the value of the coefficient of correlation between the trust and distrustful beliefs variables may be regarded as strong, and between trust and disposition to distrust may be deemed poor. Table 15 shows the obtained results.

**Table 15:** Correlations between variables trust and distrust components.

Correlations between variables	Estimate	S.E.	C.R.	P	Correlations
Trust<--> disposition to distrust (DtD)	‒.162	.060	‒2.703	.007	‒.196
Trust<--> distrustful beliefs (DB)	‒.649	.064	‒10.208	***	‒.652

*** p<0.001

By analysing the above results, one may conclude that in the case of the relationship between trust and distrustful beliefs, it is difficult to observe any regularity, and on the other hand, as regards the relation between disposition to distrust and trust, it seems that the high value of disposition to the distrust variable is more frequently related to lower values of trust.

In the next step the method of k-means clustering was used to isolate groups of similar objects (clusters). In line with the basic assumption of the method, the similarity in the cluster should be as high as possible and separate clusters should be as different as possible. Non-hierarchical methods which include k-means clustering require the assumed number of cluster to be given in advance, which strongly impacts the quality of segmentation. The application of the method allowed the isolation of two differing clusters covering 347 and 53 observations, as presented in Table 16.

**Table 16:** Descriptive statistics for both clusters in the frame of the relationship between trust and distrust components.

	N	Minimum	Maximum	Mean	Std. Deviation
**Cluster I**					
trust	347	4.13	6.50	5.4251	.47410
disposition to distrust (DtD)	347	2.00	6.00	4.0648	1.14963
distrustful beliefs (DB)	347	1.50	5.00	2.8573	.62508
**Cluster II**					
trust	53	2.13	4.00	2.7665	.49580
disposition to distrust (DtD)	53	2.00	6.00	4.6509	1.03582
distrustful beliefs (DB)	53	2.00	6.00	4.5849	1.39964

The analysis classifying cases into two clusters using the k-means clustering allows the following conclusions regarding the characteristics of each cluster:

**Cluster 1** (347 cases)—covers respondents who declared a relatively high level of trust (5.8). Moreover, the average level of distrustful beliefs is based on the to-date cooperation experience (4.1) and relatively low level of disposition to distrust resulting from internal beliefs or dispositions (2.8). Analysis of the results suggests that the respondents with a high level of trust may demonstrate very diverse levels of distrust in both categories. However, based on the obtained averages, it can be concluded that this group gained some balance between trust and distrust at concurrently relatively low disposition to distrust, which should translate into efficiency of established relations.

**Cluster 2** (combining 53 cases)–covers respondents who declared a low level of trust in relations (2.8) and medium level of distrustful beliefs (4.6) and disposition to distrust (4.6). Similarly to the first cluster, the analysis of graphical representation of the results suggests that the respondents with high levels of trust may demonstrate very diverse levels of distrust. It may be noticed, however, that the respondents in this group more often demonstrate high levels of distrust and the results are less diversified in comparison to the results obtained in the first cluster.

### 6.8. Discussion regarding the relation between interorganisational trust and distrust

The conducted analysis with the use of Pearson’s linear correlation confirmed that trust and distrust in interorganisational relations are separate but correlated constructs. The application of one of the multi-dimensional exploratory techniques, cluster analysis, allowed the identification of two clusters of respondents. The undertaken attempts to identify the relationship between constructs did not, however, lead to a clear conclusion. Both in the case of low and high trust, one may notice diverse values of distrust. Based on the obtained results, one may be tempted to assume that the disposition to distrust may be a factor reducing trust in relations. However, the relationships between constructs require more in-depth study of more diverse and numerous samples.

## 7. Conclusions

As a result of the adopted research approach, a one-dimensional scale was developed to research interorganisational trust, as well as a two-dimensional scale to study distrust in interorganisational relations. As regards interorganisational relations, the conducted research confirmed the belief that is currently shared in the literature, i.e. that trust and distrust are separate but connected dimensions. However, the existence of some ambiguity surrounding the relation between trust and distrust indicates also the need for further in-depth research.

### 7.1. Theoretical contribution

This study mainly contributes to the literature on two aspects. First, it suggests, in line with the literature on multi-level trust [75], that trust in interorganisational relationships includes relational and institutional elements potentially taking part in the creation and development of trust in interorganisational relationships. Therefore, it is important to distinguish between studies of interorganisational trust at the interpersonal level, concerning specific interorganisational relationships, and organisational level, generalised and concerning collective decision [76], using a differentiated approach to their study. According to the literature, inter-organisational trust is created on the basis of interpersonal relationships followed by norms and roles becoming institutionalised, after which trust becomes tied more to the organisation than to any specific individual [18]. The results indicate that interorganisational trust considered at the collective decision level may be a one-dimensional construct including both competence and goodwill aspects. They also suggest that trust at the interorganisational level, being a result of trust at the interpersonal level, includes generalized trust dispositions in all potential dimensions.

Secondly, our contribution to the trust-distrust literature is to affirm the co-existence of trust and distrust in relationships, responding to the call for a broader inclusion of the relationship between trust and distrust in the research agenda [24, 77]. Our research has confirmed that trust and distrust are separate constructs and that they are not at opposite ends of a continuum, and also that they may exist simultaneously [e.g. 3, 6]. Moreover, they could both be understood as an essential means of managing uncertainty in relationships [78].

### 7.2. Methodological contribution

The main research objective was to create universal scales to study trust and distrust in inter-organisational relationships. The measurement of trust was to be used, among other things, to explain strategic choices of relational strategy including value creation and appropriation [32], choice of partner [79, 80], and inter-organizational behavior, i.e. cooperation and coopetition [81, 82]. The methodological contribution of this study is therefore the development of scales measuring trust and distrust at the inter-organisational level and their empirical verification. The construction of the trust and distrust scales took into account the dimensions indicated in the literature. However, the analysis of the trust scale led to the identification of a one-dimensional solution. On the other hand, in the case of distrust, the research revealed the existence of two strongly correlated dimensions of distrust, i.e. distrustful beliefs and disposition to distrust. Due to the fact that the disposition to distrust dimension consists of only two items, it is necessary to develop this scale towards at least a three item solution [83].

### 7.3. Practical guidance for the application of the scales

Finally, it is worth pointing out some practical guidelines for the application of these scales. As indicated earlier, one may consider shortening the trust scale by omitting highly correlated items. The recommendation in this case would be to use at least four variables including T7 and T8.

Moreover, due to the definition [20, 67], which indicates that decision making about trust and establishing relationships is based on a collective mental state a larger number of respondents making decisions about the formation of relationships and their type should be examined. In our opinion this applies to both trust and distrust. Therefore we suggest that this should be 3–5 respondents in order to more accurately capture the collective mental state of the decision makers.

### 7.4. Limitations of the adopted research approach

The limitations of the adopted research approach pertain primarily to the selection of the sample. As the studies pertained to relational strategy, the selected subjects were mature enterprises and respondents included owners or top managers. In relation to the applied items, the respondents proved to be a homogeneous group by giving similar answers regarding interorganisational trust. It is worth indicating that such a concentration of non-diversified responses may affect the assessment of relations between variables. On the one hand it may suppress actual correlations (fluctuations around mean values are slight), while on the other hand respondents making assessments outside the two responses may overly affect the strength of the relationship between variables.

Perhaps, in future research the selection of the sample should be considered in terms of criteria that may diversify trust in partners. High correlations between the variables were found, as well as a high Cronbach’s alpha coefficient, e.g. on the trust scale for the study sample: 0.948. What is vital, respondents very often chose the same values in their responses (not only the same direction, which would also yield a high correlation value) for a number of variables. The consequence is a very high homogeneity of pointer variables (the same level of means and standards deviations).

This result may suggest a problem with differentiating questions regarding interorganisational trust by the respondents, which is also suggested by the absence of significant changes in Cronbach’s alpha during the removal of item—variables multiply the same information resource instead of complementing the measured variable with a new variability range. The difference in the meaning of statements, although clear for experts, might have been too subtle for respondents.

The authors dealing with the topic of trust indicate that trust is difficult to operationalise, which is caused by a multitude of theoretical approaches to this construct and the factors that may contribute to a different understanding of the same aspect of trust as the respondents, such as subjectivism, difference in perception, and cognitivism [84]. Moreover, the interorganisational trust covers two levels: an organisational level comprising collective perception and a team level. Even in the case of accurate operationalisation of the notion of trust, one still may have doubts whether two people declaring trust feel exactly the same way. Because the same score on the scale may mean something completely different for individual entities, only data from an adequately large sample can neutralise errors resulting from a different calibration of trust scales by respondents. Therefore, when drawing conclusions on the topic, cautiousness is required due to the small size of samples. Thus, one may doubt whether a reliable tool for researching trust can be created. However, this does not mean that researchers should cease attempting to do so. The limitations of the present study lead us to suggest some approaches for further research. Primarily, they indicate the need to measure interorganisational trust based on a number of diversified samples according to cultural context, industry, and type or level of the discussed relations, which would increase the possibility to generalise the obtained results. It would also be material, in this context, to repeat the research based on larger samples. Moreover, it is worth making an attempt to contrast more the variables at the stage of further research, trying at the same time to develop the scale to analyse distrust in interorganisational relations. In future research, a more comprehensive approach should be applied, based on diversified research methods including in-depth interviews and gathering data from both parties to the relationship, which will help to better understand the role of trust and distrust, as well as the relations between them.

As results from the limitations indicated above, obtaining fully satisfying measurement scales requires further work on the development of scales and replication research.

## References

[1] Cook K. S. and Schilke O. The Role of Public, Relational and Organizational Trust in Economic Affairs. Corporate Reputation Review. 2010; 13(2): 98–109.

[2] MacDuffie J. P. Inter-organizational trust and the dynamics of distrust. Journal of International Business Studies 2011; 42:35–47.

[3] Connelly B. L., Miller T., Devers C. E. Under a cloud of suspicion: Trust, distrust, and their interactive effect in interorganizational contracting. Strategic Management Journal. 2012; 33: 820–833.

[4] Lumineau F. How contracts influence trust and distrust. Journal of Management. 2017; 43:1553–1577.

[5] Seppanen R., Blomqvist K. It is not all About Trust-The Role of Distrust in Inter-Organizational Relationships. In: Collaboration Network-Centric and Frameworks Supporting. PRO-VE 2006. IFIP International Federation for Information Processing. 2006; 224. Springer, Boston, MA. 10.1007/978-0-387-38269-2_19

[6] Guo S-L., Fabrice Lumineau F. and Roy J. Lewicki R.J. Revisiting the Foundations of Organizational Distrust. Foundations and Trends in Management. 2017;1(1): pp 1–88. doi: 10.1561/3400000001

[7] Williams M., Belkin L. Y., Chen C. C. Cognitive Flexibility Matters: The Role of Multilevel Positive Affect and Cognitive Flexibility in Shaping Victims’ Cooperative and Uncooperative Behavioral Responses to Trust Violations, Group & Organization Management. 2020; 45 (2): 181–218.

[8] Cho J. The mechanism of trust and distrust formation and their relational outcomes. Journal of retailing. 2006; 82: 25–35.

[9] Molina-Morales F.X., Martínez-Fernández M.T. and Torlò V.J. The dark side of trust: the benefits, costs and optimal levels of trust for innovation performance. Long Range Planning. 2011; 44:118–133.

[10] Gargiulo M., Ertug G., 2006. The dark side of trust. In: Handbook of Trust Research. Elgar, Cheltenham, UK/Northampton, MA. 2006: 165–186.

[11] Wicks A.C., Berman S.L. and Jones T.M. Toward a conception of optimal trust: moral and strategic implications. Academy of Management Review. 1999; 24:99–116.

[12] Lewicki R., McAllister D., Bies R. Trust and Distrust: New Relationships and Realities. The Academy of Management Review, 1998; 23(3): 438–458. doi: 10.2307/259288

[13] McEvily B., Tortoriello M. Measuring trust in organisational research: Review and recommendations. Journal of Trust Research. 2011; 1 (1), 23–63.

[14] Poppo L., Zenger T. Do formal contracts and relational governance function as substitutes or complements?, Strategic Management Journal. 2002; 23(8): 707–725.

[15] Socjologia Sztompka P., Znak Kraków 2006.

[16] King B. G., Felin T. and Whetten D. A. Finding the Organization in Organizational Theory: A Meta-Theory of the Organization as a Social Actor. Organization Science. 2010; 21(1): 290–305.

[17] Zanini M. T. F., Migueles C. P. Trust as an element of informal coordination and its relationship with organizational performance. Economia. 2013; 14(2):77–87. 10.1016/j.econ.2013.08.005

[18] Schilke O., Cook K. S. A cross-level process theory of trust development in inter-organizational relationships. Strategic Organization. 2013;11(3): 281–303.

[19] Gillespie N.A. Survey measures of trust in organisational context an overview, in ed. Lyon F., Möllering G. and Saunders M., Handbook of research methods on trust, Chentelham UK, Edward Elgar. 2015.

[20] Zhong W., Su C., Peng J. Trust in inter-organizational relationships: A meta-analytic integration. Journal of Management 2017; 43(4):1050–1075.

[21] Seppänen R., Blomqvist K., Sundqvist S. Measuring inter-organizational trust—a critical review of the empirical research in 1990–2003, Industrial marketing management. 2007; 36 (2): 249–265.

[22] Park S. and Kim E.J. Fostering organizational learning through leadership and knowledge sharing. Journal of Knowledge Management. 2018; 22(6):1408–1423, doi: 10.1108/JKM-10-2017-0467

[23] Bachmann R., Zaheer A. Handbook of Trust Research, Edward Elgar Publishing Limited, Cheltenham, UK, Northampton, MA. 2006.

[24] Raza-Ullah T., Kostis A. Do trust and distrust in coopetition matter to performance? European Management Journal. 2020; 38 (3): 367–376.

[25] Burke C.S., Sims D.E., Lazzara E.H., Salas E. Trust in leadership: a multi-level review and integration. Leadership Quarterly. 2007; 18(6): 606–632.

[26] Fang S.C., Yang C.W. and Hsu W.Y. Inter-organizational knowledge transfer: the perspective of knowledge governance. Journal of Knowledge Management. 2013;17 (6): 943–957. doi: 10.1108/JKM-04-2013-0138

[27] Savolainen T., Lopes- Fresno P. Trust as intangibleasset: Enablingintelectual capital developmentby leadershipfor vitalityand innovativeness. Electric Journal of Knowledge Management.2013;11 (3):244–255.

[28] Mayer R., Davis J., Schoorman F. An integrative model of organizational trust. Academy of Management Review.1995; 20(3): 709–734.

[29] Tzafrir S.S., Eitam-Meilik M. The impact of downsizing on trust and employee practices in high tech firms: a longitudinal analysis. Journal of High Technology Management Research.2005;16:193–207.

[30] Currall S. C. and Inkpen A. C. Strategic Alliances and the Evolution of Trust across Levels, in West M. A., Tjosvold D. and Smith K. G. (eds) International Handbook of Organizational Teamwork and Cooperative Working. 2003: 533–49. New York: Wiley.

[31] Zaheer A., McEvily B., Perrone V. Does trust matter? Exploring the effects of interior ganizational and interpersonal trust on performance. Organization Science. 1998; 9(2): 141–159.

[32] Vanneste B. S. From interpersonal to interorganisational trust: The role of indirect reciprocity, Journal of Trust Research. 2016; 6(1): 7–36, doi: 10.1080/21515581.2015.1108849

[33] Blomqvist K. Partnering in the dynamic environment: The role of trust in asymmetric partnership formation, doctor of science thesis. 2002 Lappeenranta University of Technology, Lappeenranta.

[34] Gulati R. Does familiarity breed trust? The implications of repeated ties for contractual choice inalliances. Academy of Management Journal. 1995; 38 (1): 85–112.

[35] Lavie D., Haunschild P., Khanna P. Organizational differences, relational mechanisms, and alliance performance, Strategic Management Journal. 2012; 33 (13): 1453–1479.

[36] Koźmiński A.K., Latusek-Jurczak D. (ed.) Relacje międzyorganizacyjne w naukach o zarządzaniu. 2014. Oficyna a Wolters Kluwer business, Warszawa.

[37] Chang Y-S., Fang, S-R., Antecedents and distinctions between online trust and distrust: Predicting high- and low-risk internet behaviours. Journal of Electronic Research 2013; 14 (2): 149–166.

[38] Priem R.L. and Nystrom P.C. Exploring the dynamics of workgroup fracture: common ground, trust-with-trepidation, and warranted distrust, Journal of Management. 2014; 40 (3):764–795. doi: 10.1177/0149206311412191

[39] Janowicz-Panjaitan M. Noorderhaven N. G. Trust, calculation, and inter-organizational learning of tacit knowledge: An organizational roles perspective. Organization Studies 2009; 30:1021–1044.

[40] Welch M. Rethinking relationship. Management, exploring the dimension of trust, “Journal of Communication Management. 2006; 10(2):138–155.

[41] Swift T. Trust, reputation and corporate accountability to stakeholders. Business Ethics. A European Review. 2001;10(1):16–26.

[42] Govier T. Is it a jungle out there? Trust, distrust and the construction of social reality. Dialogue. 1994; 33 (2):237–252.

[43] Dirks K. T., Lewicki R. J., Zaheer A. Repairing relationships within and between organizations: Building a conceptual foundation. *The Academy of Management Review*. 2009; 34 *(*1), 68–84. 10.5465/AMR.2009.35713285

[44] Moody G.D., Galletta D.F. and Lowry P.B. When trust and distrust collide online: the engenderment and role of consumer ambivalence in online consumer behavior. Electronic Commerce Research and Applications. 2014;13 (4): 266–282, doi: 10.1016/j.elerap.2014.05.001

[45] McKnight D.H., Chervany N.L. Trust and distrust definitions: One bite at a time [in:] Trust in cyber-societies. 2001. Springer Berlin Heidelberg.

[46] Fein S. Effects of suspicion on attributional thinking and the correspondence bias, Journal of Personality and Social Psychology.1996;70:1164–1184.

[47] Rafaeli A., Sagy Y., and Derfler-Rozin R. Logos and initial compliance: A strong case of mindless trust. Organization Science. 2008; 19:845–859.

[48] Colquitt J.A., Scott B.A., LePine J.A. Trust, Trustworthiness, and Trust Propensity: A Meta-Analytic Test of Their Unique Relationships With Risk Taking and Job Performance. Journal of Applied Psychology. 2008; 92(4):909–927. 10.1037/0021-9010.92.4.909 17638454

[49] Ellonen R., Blomqvist K., Puumalainen K. The role of trust in organizational innovativeness. European Journal of Innovation Management. 2008; 11(2):160–181.

[50] McKnight D.H.; Choudhury V. Distrust and trust in B2C e-commerce: Do they differ? ICEC ’06: Proceedings of the 8th international conference on Electronic commerce: The new e-commerce: innovations for conquering current barriers, obstacles and limitations to conducting successful business on the internet. August 2006;482–491.

[51] Şengün A.E. Wasti S.N. Trust types, distrust, and performance outcomes in small business relationships: the pharmacy–drug warehouse case, The Service Industries Journal. 2011; 31:2, 287–309, doi: 10.1080/02642060902759137

[52] Schoorman F. D., Mayer R. C., and Davis J. H. An integrative model of organizational trust: Past, present, and future. Academy of Management Review 2008; 32: 344–354.

[53] Benbasat I., Gefen D., and Pavlou P. A. Introduction to the special issue on novel perspectives on trust in information systems. MIS Quarterly. 2010; 34 (2): 367–371.

[54] Dimoka A. What does the brain tell us about trust and distrust? Evidence from a functional neuroimaging study. MIS Quarterly 2010; 34(2):373–396.

[55] Ullman-Margalit E. Trust, distrust and the in-between. In: Hardin R. (Ed.), Distrust. 2004. New York: Russell Sage Foundation.

[56] Parkhe A., Miller S. R. The structure of optimal trust: A comment and some extensions. Academy of Management Review. 2000; 25:10–11.

[57] Mozuni M., Jonas W. An introduction to the morphological Delphi Method for design: A tool for future-oriented design research. The Journal of Design, Economics, and Innovation. 2018; 3(4): 303–318.

[58] Podsakoff P.M.,. MacKenzie S. B., Podsakoff N.P. Sources of Method Bias in Social Science Research and Recommendations on How to Control It. Annual Review of Psychology. 2012; 63:1, 539–569. 10.1146/annurev-psych-120710-100452 21838546

[59] Kock N. Common method bias in PLS-SEM: A full collinearity assessment approach. International Journal of e-Collaboration. 2015;11(4): 1–10.

[60] Shapiro S. P. The Social Control of Impersonal Trust, American Journal of Sociology. 1987; 93 (3):623–658.

[61] Ganesan S. Determinants of long-term orientation in buyer-seller relationships. Journal of Marketing. 1994; 58 (2): 1–19.

[62] Chow S., Holden R. Toward an Understanding of loyalty: The Moderating Role of Trust. Journal of Managerial Issues. 1997; 9(3): 275–298.

[63] Doney P.M. and Cannon J.P. An Examination of the Nature of Trust in Buyer-Seller Relationships. Journal of Marketing.1997; 61:35–51. 10.2307/1251829

[64] Pavlou P.A., Tan Y.H., Gefen D. The transitional role of institutional trust in online inter-organizational relationships, Proceedings of the 36^th^ Annual Hawaii International Conference on System Sciences, IEEE, 2003:1–10.

[65] Lui S. S., Ngo H. The role of trust and contractual safeguards on cooperation in non-equity alliances. Journal of Management. 2004; 30: 471–485.

[66] Jiang X., Jiang F., Cai X., Liu H. How does trust affect alliance performance? The mediating role of resource sharing. Industrial Marketing Management. 2015;45(1): 128–138.

[67] Czernek K., Czakon W. Trust-building processes in tourist coopetition: The case of a Polish region. Tourism Management. 2016; 52: 380–394.

[68] Grover V., Malhotra M.K. Transaction cost framework in operations and supply chain management research: Theory and measurement. Journal of Operations Management. 2003; 21 (4): 457–473.

[69] Moody G. D., Lowry P. B., Galletta D. F. It’s complicated: Explaining the relationship between trust, distrust, and ambivalence in online transaction relationships using polynomial regression analysis and response surface analysis. European Journal of Information Systems. 2017; 26(4), 379–413. doi: 10.1057/s41303-016-0027-9

[70] Bowling A. (2002). Research methods in health: investigating health and health services. Open University Press, Buckingham, Filadelfia, p.131.

[71] Fornell C., & Larcker D. F. (1981). Evaluating structural equation models with unobservable variables and measurement error. Journal of Marketing Research, 18(1), 39–50.

[72] Krabbe P.F.M. The Measurement of Health and Health Status: Concepts, Methods and Applications from a Multidisciplinary Perspective. 2017; Academic Press.

[73] Mollering G. Perceived trustworthiness and inter-firm governance: Empirical evidence from the UK printing industry. Cambridge Journal of Economics. 2002; 26(2), 139–160.

[74] Kline R.B. Convergence of Structural Equation Modeling and Multilevel Modeling in. ed. Williams M. Handbook of methological innovation. 2011; Sage Thousand Oaks, C.A.

[75] Bagozzi R.P., Yi Y., Phillips L.W. Assessing construct validity in organizational research. Administrative Science Quarterly. 1991; 36: 421–458.

[76] Nielsen B.B. Trust in strategic alliances: Toward a co-evolutionary research model. Journal of trust research. 2011; 1(2):159–176.

[77] Vanneste B.S., Puranam P., Kretschmer T. Trust over Time in Exchange Relationships: Meta-analysis and Theory. Strategic Management Journal. 2014; 35(12): 1891–1902.

[78] Saunders M. N., Dietz G., Thornhill A. Trust and distrust: Polar opposites, or independent but co-existing? Human Relations. 2014. 67(6): 639–665.

[79] Garcia‐Castro R., Aguilera R. V. Incremental value creation and appropriation in a world with multiple stakeholders. Strategic Management Journal. 2015; 36(1): 137–147.

[80] Zakrzewska-Bielawska A., Lewicka D. (2021), A company’s relational strategy: Linkage between strategic choices, attributes, and outcomes, PLOS ONE 16(7): e0254531, doi: 10.1371/journal.pone.0254531 34293001 PMC8297868

[81] Malhotra N. K., Agarwal J. A stakeholder perspective on relationship marketing: framework and propositions. Journal of Relationship Marketing. 2002; 1(2): 3–37.

[82] Lewicka D., Zakrzewska-Bielawska A. (2020). Interorganizational Trust in Business Relations: Cooperation and Coopetition. In Zakrzewska-Bielawska A., Staniec I. (Eds.). Contemporary Challenges in Cooperation and Coopetition in the Age of Industry 4.0 2020; 155–174.

[83] Konarski R. 2009, Modele równań strukturalnych. Teoria i praktyka, PWN, Warszawa.

[84] Piórkowska K. Cognitive and Methodological Content of Social and Cognitive Psychology in the Context of Management Science. Research Papers of the Wroclaw University of Economics. 2014; 340: 112–120.

